# Supplementary material for: Seroprevalence of hepatitis B virus among pregnant women attending Antenatal care in Dilla University Referral Hospital Gedio Zone, Ethiopia; health facility based cross-sectional study
Source: PLoS One. 2021 Mar 25;16(3):e0249216. doi: 10.1371/journal.pone.0249216 (PMC7993874; doi:10.1371/journal.pone.0249216)
Supplement: S1 Questionnaire — (PDF) [file pone.0249216.s001.pdf]

A. **Survey questionnaire (English version)**

Sero-prevalence and associated risk factor of HBV among pregnant women attending ANC in DURH

Questionnaire Number___ Date of interview____________________

Participant Code___________________

Pregnant registration number__________________

S No Questions Response

100 Socio-demographic information (Circle One answer)

101 Age (in years) ______________

102 religion______________

103 ethnicity ______________

104 Marital status? a. Single b. Married c. Widowed d. Divorced

105 Education Level? a. No formal education at all b. Primary education

c. Secondary education d. College level

106 What is your occupational status? a. Self-employed b. Government employed c. Private employed d. Not employed

105 7Pregnancy States? a. First b. Second c. Third d. Four & above

200 factors associated with HBV transmission

201 History of multiple sexual partner 1. YES 2. NO

202 History of blood transfusion 1. YES 2. NO

203 History of Tattooing 1.YES 2. NO

204 sharp material sharing with others 1.YES 2. NO

205 did you have any surgical procedure 1. YES 2. NO

206 did you have any genital mutilation 1. YES 2. NO

207 did you have any abortion before 1. YES 2. NO

208 Is there any history of jaundiced patient 1. YES 2. NO

209 do you have screening for HBV 1. YES 2. NO

210 do you have vaccination for HBV 1. YES 2. NO

300 Serology (Laboratory findings for HBsAg) 1. Positive 2. Negative

301 HIV sero status 1. Positive 2. Negative

B. Survey questionnaire (Amharic version)

የመረጃመሰብሰቢያመጠይቅፎርም (ቅፅ) ዲላ ዩኒቨርሲቲ ህክምና ት/ቤት በጥናቱተሳታፊበሆኑግለሰቦችየሚሞላፎርም

መጠይቅየተወሰደበትቀን ____________________የተሳታፊውቁጥር __________

የክትትልመለያቁጥር_________

100 የግለሰቡአካላዊማህበራዊእናኢኮኖሚያዊዝርዝር

101 ዕድሜ ________________________________

102 ሀይማኖት________________________________

103 ብሄር________________________________

104 የጋብቻሁኔታ? 1. ያላገባ 2. ያገባ 3. የትዳርጓደኛ የሞተበት 4. የተፋቱ

105 የትምህርትሁኔታ?

1. ያልተማረ

2. የመጀመሪያደረጃት/ትያጠናቀቀ

3. ሁለተኛደረጃት/ትያጠናቀቀ

4. የኮሌጅተማሪ

106 ስራ? 1. የግል 2. የመንግስት 3. የቤትእመቤት 4. ሥራአጥ

107 የእርግዝናሁኔታ? 1. የመጀመሪያ 2. ሁለተኛ 3. ሶስተኛ 4. አራተኛናከዚያበላይ

200 ሄፐታይተስ “ቢ”ከሱ ጋር የተያያዙ ተጋሊጪ መንስኤዎች

201 በህይዎትሆ ከብዙ ሰው ጋር ጥንቃቄ የጎደሆው ግብረሥጋ ግኑኝነት አድርገዋ?

1አወ 2 የለም

201ደምና የደም ዉጤቶችን ወስደው ያዉቃለ1አወ 2 የለም

202ቀደ-ጥገና አጋጥሞዎት 1አወ 2 የለም

203 ተገርዘዋል 1አወ 2 የለም

204 ስለታማ ነገሮችን ከሌሎች ጋር ይጋራሉ 1አወ 2 የለም

205 ተነቅሰዋል 1አወ 2 የለም

206 ተገርዘዋል 1አወ 2 የለም

207ውርጃአጋጥሞዎት1አወ 2 የለም

208 አይኑ ቢጫ የሆነ ሰዉ ጋር ተነካክተዋል 1አወ 2 የለም

209የሄፓታይቴስ ቢ ቫይረስ ተመርምረዋል 1አወ 2 የለም

210 የሄፓታይቴስ ቢ ቫይረስ ክትበት ተከትበዋል? 1አወ 2 የለም

300የሄፓታይቴስ ቢ ቫይረስ . የደምምርመራውጤት   1. ፖዘቲብ   2. ነጋቲብ

301 የኤችየቪ ቫይረስ የደምምርመራውጤት   1. ፖዘቲብ   2. ነጋቲብ
