# Supplementary material for: Seroprevalence of hepatitis B virus among pregnant women attending Antenatal care in Dilla University Referral Hospital Gedio Zone, Ethiopia; health facility based cross-sectional study
Source: PLoS One. 2021 Mar 25;16(3):e0249216. doi: 10.1371/journal.pone.0249216 (PMC7993874; doi:10.1371/journal.pone.0249216)
Supplement: S1 Table — (PDF) [file pone.0249216.s002.pdf]

S1 Table; Seroprevalence of HBsAg among pregnant women attending ANC in DURH from December to May 28, 2017

Options	Frequency	Percent
Positive	11	5.1%
Negative	204	94.9%
