# Supplementary material for: Seroprevalence of hepatitis B virus among pregnant women attending Antenatal care in Dilla University Referral Hospital Gedio Zone, Ethiopia; health facility based cross-sectional study
Source: PLoS One. 2021 Mar 25;16(3):e0249216. doi: 10.1371/journal.pone.0249216 (PMC7993874; doi:10.1371/journal.pone.0249216)
Supplement: Attachment — Submitted filename: Rebuttal Letter.pdf [file pone.0249216.s003.pdf]

Manuscript PONE-D-20-36836

Point- by- Point Rebuttal Letter

We really thank the editor and the two reviewers for their valuable comments on our manuscript.

Please kindly find below our response to each point raised by the academic editor and reviewers. We hope that we clearly addressed all of them, and that the manuscript will be now suited for publication. We bolded the comments, and highlighted the responses by water blue color.

Sincerely,

On behalf of all the four authors,

Worku Ketema Bededa

Academic editor:

Journal requirements

1. **Plos one templates**
   - ✓ We have checked the templates and made the adjustments to meet the journal requirements.
2. **Additional information regarding survey or questionnaire-**
   - ✓ We put the questionnaire in the supplementary data in both Amharic and English and please kindly see the supplementary documents.
3. **State whether you validated the questionnaire prior to testing on the study participants. Please provide details regarding the validation groups within the method section**

- ✓ Thank you for your comments; The questionnaire was first prepared in English and translated back to Amharic then translated back to English to ensure consistency of the questions. Pre-testing was done on 12 pregnant mother prior to the study (5 % of the total sample size, which was 236). The clarity, understandability and flow of each question and the time to fill the questionnaire were assessed with a bit modification of the questionnaire. All the collected data was checked for completeness by the principal investigator daily. All the data were double entered to ensure the data quality

4. **In your methods; Please provide additional information about the participant recruitment method and the demographic details of your participants. Please ensure you have provided sufficient details to replicate the analysis such as;**

-A description of any inclusion/exclusion criteria that were applied to participant recruitment

-A table of relevant demographic details

-A statement as to whether your sample can be considered as representative of a large population

- Description of how participants were recruited, and

-Description of where participants were recruited and where the research took place

Response; We are grateful to respond to your constructive comments, and here are the responses one by one

- ➢ Regarding the Eligibility criteria

Inclusion Criteria

- All Pregnant women were attending antenatal clinic in the DURH during the study period and who were volunteering and give informed consent

Exclusion Criteria

- Those women who were unable to communicate due to any problem

- The table of relevant demographic details has been incorporated in the main manuscript(Table 1)
- One of the limitations of this paper is small sample size(due to financial constraint by the time of the study), and I admit that the finding will not be an exact representative of large population, but will only give as an insight on the burden of the virus and associated factors. It will pave a path for the future study that will be done with better sample size.
- Participant recruitment has been elaborated under the sampling procedure. Participants were recruited from the ANC clinic of Dilla University Referral Hospital, Dilla, Ethiopia

Sampling procedure

- We appreciate for your review, and here is the response for this specific comment; Study subjects were selected by systematic sampling method by dividing the sample size by the number of pregnant women attending ANC two months before the study period. They were permitted to enter into the ANC clinic room for their routine follow up based on their turn of registration one by one. The aim of the study was briefed to the subjects, and they were asked for their willingness to be interviewed. Those who met the inclusion criteria and volunteer were included. Pregnant women who attended the ANC clinic for more than one time during the study period were excluded.

Study variables-We are sorry for not to incorporate this in the original manuscript initially,and here are the edited one;

Dependent variables

- The prevalence of Hepatitis B surface Antigen, among pregnant women attending ANC in DURH, southern Ethiopia

Independent variables

- Socio demographic variables like;

1. Maternal age marital status,
2. Occupational status
3. Educational status
4. Any surgical procedure
5. Gestational age

6. Body tattooing
7. Genital mutilation
8. History of blood transfusion
9. History of multiple sexual practices

6. Table as part of main manuscript and Supplementary table- Thank you for the comments and we have made corrections.

7. Title differences- Thank you, we edited it

Reviewer #1

Comment 1

Abstract

Methods; the authors should include the inclusion and inclusion criteria in the abstract. What were the outcome measures?

Response 1; the reviewer has made interesting points, and we made an amendments.

- All Pregnant women who were attending antenatal clinic and were volunteer during the study period were included, whereas those women who were unable to communicate due to any problem and not volunteer to give informed consent were excluded.

Comment 2; **the authors wrote; 215 study participates were tested for HIV antibody, of which 10 (4.5%) were turned to be positive for HIV. 4 (1.86 %) of the study participants who were tested for HIV were also positive for HBsAg. The authors should avoid starting a sentence with figures.**

Response 2; we thank the reviewer for his assessment, and we edited is as below

- Among the study participants, 91.1 % (215) were tested for HIV antibody during the ANC visit, with the positivity rate of 4.5 %. The result showed 1.86 % of the study participants who were tested for HIV were also positive for HBsAg.

Comment 3; **the authors stated the Seropositivity of Hepatitis B Virus among Pregnant Women was found to be significant. Why did they say that the study is significant? How did they arrive at significant?**

Response 3; we said it was significant in our conclusion, after comparing our result with the WHO established criteria 2015(2).

Comment 4; the **authors should beef up the justification for the study**

Response 4; we thank the reviewer for constructive comments,

- Some amendments have been done, at the last paragraph of the introduction. We especially interested to quote the statement word by word, "Although there are guidelines for universal infant HBV vaccination, rates of maternal HBV infection have increased annually by 5.5% since 1998" (10).We ,hence interested to investigate the burden of Seropositivity in the specific study area, and put the path for the possible interventions if needed.

Reviewer # 2

Comment 1

In the introduction part; the authors should clearly summarize the state of research in the field by citing more recent seroprevalence studies, globally. From JAMA, WHO…

Response 1; we understand and agree with this observation, and we thank the reviewer for pointing this out. We incorporated the latest publications on the seroprevalence, among which the one published on JAMA. We also added the recent studies done globally and locally like studies done in Gambia, Ghana, Southern Ethiopia, and Northern Ethiopia, included in the introduction and discussion parts.

Comment 2; in the material and method, the sample size calculation procedure is not easy to read and understand, therefore it should be elucidated

Response 2; we apologise for the inconveniences and we hope know you will get the documents more visible

Sample was calculated by taking overall Hepatitis B infection prevalence among a cross-sectional study conducted among pregnant women in Gondar in 2008. Of 209 mothers included in the study, 5.3% 3.0% level of significance / margin of error (23). .This sample size will be estimated using the formula for calculating sample size for cross sectional study of estimation a single population proportion as described below.

$n = (Z\alpha/2)^2 \; P\,(1\text{-}P)/\,(d)^{\,2}$

$= (1.96)^2 0.053(1\text{-}0.053)/\,(0.03)^2$

214.3~214

Assumptions

Z =Standard deviation of the normal distribution = 1.96 (confidence level at 95%)

P = prevalence 5.3 %(a cross-sectional study was conducted among pregnant women in Gondar in 2008.

100-P = pregnant women who not exposed

d = Tolerable error / level of significance = 3.0%.

X=10 % non-respondent rate = 21.43

Sample size = n (Minimum sample size) + X (non- respondent) Sample size (N) = 214 + 22 = 236. Sample size was 236

Comment 3; Result-The results are not clearly presented, some values are missing and some values are different for the one in the tables. Overall the author should pay the attention to rephrase the results in appropriate manner. A table summarizing the sociodemographic characteristics should also be added, while the other tables could also be restructured.

Response 3; we thank the reviewer for pointing out this inconsistency. It is now corrected as showed below.

Table 1 Socio-demographic characteristics of pregnant women attending antenatal care at at DURH from December to May 28, 2017

Variable	Option	Total no%
Age in years	16-20	38(17.6%)
	21-25	93(43.1%)
	26-30	73(33.8%)
	31-above	11(5.1%)
Educational status	illiterate	45(20.8%)
	Primary	100(46.3%)
	Secondary	54(25.8%)
	College	16(7.4%)
Occupation	unemployed	9(4.2%)
	House wife	142(65.07%
	governmental	28(13%)
	Private	35(16.7%)
Ethnicity	Gedio	100(46.5%)
	Wolyta	38(17.6%)
	gurage	25(11.6%)
	amhara	18(8.3%)
	others	34(15.7%)
Religion	protestant	148(68.5%)
	orthodox	45(20.8%)
	Muslim	20(9.3%)
	Others	2(0.9%)

Comment 4; **Discussion-The authors should site the original studies from USA and India (mentioned in the discussion), and enrich the studies with more recent publications.**

Response 4; we thank the reviewer for his kind comments and useful insights.

We tried to incorporate the recent publications on the specific topic, and also reviewed and incorporated the original studies done in USA and India that have mentioned in the original manuscript. In this study, the prevalence of Seropositivity for Hep B virus was found to be 5.1%, which was higher than research conducted in the United States to determine the seroprevalence of hepatitis B surface antigen in pregnant women. HBsAg prevalence among white non-Hispanics was 0.60%, black non-Hispanics 0.97%, Hispanics 0.14%, and Asians 5.79% (20) .It also much higher than another study conducted in India in 2016, which was 1.01 % (21) .In this study, most of the participants were in the second gravidity 90(41.9%) which was comparable to research conducted in the United States .

The Seropositivity rate in this study is also lower than the recent studies done in Gambia, Ghana, Southern and Northern parties of Ethiopia, which were 9.2 %, 7.7%, 7.3 % and 9.2 % respectively. (17-20)

Comment 5; since the authors mother tongue is not English, they should carefully revise the manuscript for grammatical and syntactical errors

Response 5; We admit that, and tried to correct some grammatical errors with the help of my fellow English department friends, and I hope you will get it better than the previous documents.
