# Supplementary material for: Seroprevalence of hepatitis B virus among pregnant women attending Antenatal care in Dilla University Referral Hospital Gedio Zone, Ethiopia; health facility based cross-sectional study
Source: PLoS One. 2021 Mar 25;16(3):e0249216. doi: 10.1371/journal.pone.0249216 (PMC7993874; doi:10.1371/journal.pone.0249216)
Supplement: Attachment — Submitted filename: Letter to Reviewers on Minor revision on HBV among pre.pdf [file pone.0249216.s004.pdf]

Manuscript PONE-D-20-36836R1

Point- by- Point Rebuttal Letter

We really thank the academic editor, and the reviewers for their valuable comments on our manuscript.

Please kindly find below our response to each point raised by the academic editor and reviewers. We hope that we clearly addressed all of them, and that the manuscript will be now appropriate for publication. We bolded the comments, and highlighted the responses by green color.

Sincerely,

On behalf of all the four authors,

Worku Ketema Bededa

Journal Requirements:

1.) **Please review your reference list to ensure that it is complete and correct.**
   - Thank you for your guide, and we have checked the templates and our references list is in accordance with PLOS.

Reviewer #1: **No comments.**

Response: we are grateful for addressing all your previous comments!

Reviewer #2: We thank the authors for adequately studying and addressing both the Editor's and our comments. The manuscript is now closer to meeting PLOS publication criteria, but there are still some points that can be mended.

Abstract and Introduction: there are some minor grammatical mistakes and some parentheses that are out of place that could easily be corrected.

Response: the reviewer has made interesting points, we appreciate your detail evaluation of the paper, and we now corrected the raised comments.

Material and Method: Thank you for pointing out the inclusion and exclusion criteria and for elucidating the sample size calculation. In the section 2.6.2 Independent variables, maternal age and marital status should be in different lines, as they represent two separate sociodemographic characteristics.

Response: Thank you for your valuable observation, and we made correction.

Results: The authors should especially pay attention at further correcting section 3.3. For example, they state "955 CI" or "95 CI" where there should be 95% CI. In the text they are referring to sharp material sharing with others, while there are no relevant data in Table 2. Concerning genital mutilation, the p-value is different in the text and in Table 2. Also, the last line from Table 2, HIV status- Option No, is missing.

Response: we thank the reviewer for his kind comments and useful insights. The edition and incorporation of the missing values have been made.

Discussion: There are some parentheses here also that are misplaced.

The authors specifically state "In this study multi-partners, gravidity and

HIV status had significant association", while earlier, in the results, they state that "Among those factors affecting the transmission of HBV infection like age, marital status, gravidity, educational level, religion, and ethnicity none has a significant association". Since gravidity was not found to have a significant association with HBsAg status, this sentence should be corrected.

Finally, when the authors say that HIV and HBV share the same mechanisms of transmission, they should cite relevant bibliography.

Response: we are grateful to respond to your constructive comments, and accordingly edition has been made. Regarding the last comment, we included the references with great honor. [29-33]

We finally want to acknowledge both the reviewers for their detailed and valuable comments, and we hope now our manuscript is ready for publication on this esteemed journal.
